# THz ATR-TDS Spectroscopy of Acetone–Water Mixtures: Hydrogen Bonding to Dipole–Dipole Dynamics

**DOI:** 10.3390/ijms27125188

**Published:** 2026-06-08

**Authors:** Zahra Mazaheri, Anagha Ramankandath, Junaid Yaseen, Can Koral, Gian Paolo Papari, Antonello Andreone

**Affiliations:** 1 Department of Physics “E. Pancini”, University of Naples “Federico II”, 80126 Naples, Italy; junaid.yaseen@unina.it (J.Y.); gianpaolo.papari@unina.it (G.P.P.); 2 Department of Health Sciences, University of Basilicata, 85100 Potenza, Italy; can.koral@unibas.it; 3 School of Photonics, Cochin University of Science and Technology, Cochin 682022, India; anagharmadhu2002@gmail.com; 4 Naples Research Unit, National Institute for Nuclear Physics (INFN), 80126 Naples, Italy

**Keywords:** terahertz time-domain spectroscopy, attenuated total reflection, hydrogen bond dynamics, acetone–water mixtures

## Abstract

Attenuated total reflection time-domain spectroscopy (ATR-TDS) in the terahertz regime was employed to investigate the dielectric response of water–acetone mixtures over the full molar concentration range. The ATR configuration enabled stable measurements in a controlled and nearly closed environment, minimizing acetone evaporation and allowing reliable characterization of this highly volatile binary system. The complex dielectric function, retrieved in the 0.4–1.6 THz range, was analyzed by means of a double Cole–Cole model, which provided a more consistent description of the mixtures than a simple Debye-based approach. A strongly nonlinear dependence on composition was observed, with the highest sensitivity in the water-rich region, where even small amounts of acetone produced a marked change in both the real and imaginary parts of the dielectric function. The extracted parameters indicate that acetone primarily suppresses the slow, cooperative relaxation channel associated with the hydrogen-bond network of water, whereas the faster channel remains comparatively less affected, consistent with its more local intermolecular origin. The evolution of the Kirkwood–Fröhlich correlation factors and of the broadening parameters further supports a progressive transition from a highly correlated hydrogen-bonded liquid to a structurally heterogeneous and weakly cooperative dipolar environment. These results demonstrate that THz ATR-TDS is a sensitive tool for probing intermolecular reorganization in aqueous binary mixtures, providing a physically grounded framework for the detection of acetone and other volatile hydrogen-bond-active species in water-based systems.

## 1. Introduction

Acetone (${\mathrm{CH}}_{3}$${\mathrm{COCH}}_{3}$) is the smallest and one of the most widely used ketones. Because of its complete miscibility with water and many other protic and aprotic solvents, it is extensively employed in industrial processes, laboratory practice, and chemical synthesis. At the same time, its widespread use makes it a common environmental contaminant, as it may enter natural waters through industrial discharge, solvent misuse, accidental spills, or atmospheric deposition. Acetone is also relevant in biological systems, where it is produced during fatty acid metabolism, and its concentration can increase under physiological or pathological conditions such as fasting, prolonged exercise, and uncontrolled diabetes [1,2]. Monitoring acetone in aqueous media is therefore of interest not only for environmental analysis but also for biomedical and diagnostic applications.

From a molecular perspective, acetone is an aprotic polar solvent. Although it cannot donate hydrogen bonds, it can efficiently accept O–H hydrogen bonds through the lone pairs of its carbonyl oxygen [3,4,5]. When dissolved in water, acetone perturbs the three-dimensional hydrogen bond network that characterizes the liquid state of water, thereby modifying both the local solvation environment and the collective intermolecular dynamics [6,7]. Even at relatively low concentrations, this perturbation may induce substantial changes in dipolar correlations, hydrogen bond connectivity, and molecular relaxation processes [5]. A detailed spectroscopic investigation of water–acetone mixtures is therefore important not only for assessing concentration-dependent dielectric properties but also for understanding the microscopic reorganization of the liquid structure.

The terahertz (THz) spectral range is particularly well-suited for this purpose because it probes intermolecular motions and dielectric relaxation processes occurring on picosecond and sub-picosecond timescales. In polar liquids, THz spectroscopy is sensitive to collective molecular fluctuations, dipole–dipole correlations, and transient rearrangements of hydrogen bond networks, making it a powerful tool for investigating aqueous systems, hydration dynamics, and solute–solvent interactions [8,9,10,11]. In water-based mixtures, the THz dielectric response is not simply determined by the intrinsic properties of the pure components but rather by the way in which intermolecular interactions reorganize the liquid structure as a function of composition.

The experimental investigation of acetone–water mixtures in the THz domain is, however, not straightforward. On the one hand, water is strongly absorbing in this frequency range, which makes conventional transmission measurements difficult. On the other hand, acetone is highly volatile, and even modest evaporation during acquisition may alter the actual composition of the mixture, especially in the water-rich regime, where the spectroscopic response is expected to be most sensitive to small concentration changes. These constraints motivated the use of attenuated total reflection (ATR) combined with terahertz time-domain spectroscopy (TDS). In the ATR geometry, the sample is probed through the evanescent field generated at the prism–sample interface, which reduces the effective interaction length and enables the characterization of highly absorbing liquids while minimizing uncertainties related to sample thickness and evaporation. When coupled with coherent TDS detection, ATR also allows the retrieval of both the amplitude and phase of the reflected electric field and therefore of the complex dielectric response of the liquid [12,13,14].

In this work, we employed ATR-TDS to investigate water–acetone mixtures over the full molar concentration range in the frequency window of 0.4–1.6 THz. The aim is to clarify how the progressive addition of acetone modifies the dielectric response and intermolecular relaxation dynamics of the system, with particular attention to the transition from a highly cooperative hydrogen-bond-dominated network to a more weakly correlated dipolar liquid. Although the present study focuses on acetone, the broader physical issue addressed here is the reorganization of water-rich hydrogen bond networks upon the addition of a polar solute. In this sense, acetone should not be viewed as uniquely special but rather as a particularly relevant aprotic hydrogen bond acceptor that provides a well-defined model system for investigating how solute-induced perturbations reshape collective water dynamics. The results presented here should therefore be positioned within the wider context of recent studies on aqueous mixtures and hydrogen bond network reorganization [10,15,16,17,18].

## 2. Results and Discussion

A set of water–acetone mixtures spanning the full composition range, from pure water to pure acetone, were investigated in order to track the evolution of the dielectric response as a function of the acetone molar fraction. As a first step, the reliability of the THz ATR-TDS approach was assessed by measuring the dielectric function of the two pure liquids, whose behavior in the THz range provided a useful reference for the subsequent analysis of the mixtures.

For pure water, the dielectric response in the terahertz region is governed by two characteristic relaxation processes: a slow one, $\tau_{1}$, generally associated with collective reorientation of molecular dipoles within the hydrogen-bond network, and a faster one, $\tau_{2}$, related to more localized intermolecular motions, including short-range hydrogen bond rearrangements and vibrational or librational dynamics [19,20]. In this case, the dielectric response is commonly described by a double Debye model:

(1) $$\tilde{\varepsilon }\left(\omega \right)=\varepsilon_{\infty }+\frac{\Delta \varepsilon_{1}}{1+i\omega \tau_{1}}+\frac{\Delta \varepsilon_{2}}{1+i\omega \tau_{2}}$$

where $\varepsilon_{\infty }$ is the high-frequency permittivity, and $\Delta \varepsilon_{1}$ and $\Delta \varepsilon_{2}$ are the dielectric strengths of the two relaxation channels.

The Debye formalism assumes discrete relaxation events characterized by single time constants in a statistically homogeneous environment. While this approximation is often adequate for pure water in the THz range, it becomes less satisfactory for acetone and, even more, for water–acetone mixtures, where overlapping intermolecular contributions, hydrogen-bond disruption, and local structural heterogeneity are expected to produce non-Debye behavior [8,18]. For this reason, a broader phenomenological description is required.

To account for all these effects, the dielectric spectra were analyzed using a double Cole–Cole model [21]:

(2) $$\tilde{\varepsilon }\left(\omega \right)=\varepsilon_{\infty }+\frac{\Delta \varepsilon_{1}}{{\left(1+i\omega \tau_{1}\right)}^{1-\alpha_{1}}}+\frac{\Delta \varepsilon_{2}}{{\left(1+i\omega \tau_{2}\right)}^{1-\alpha_{2}}}$$

where $\alpha_{1}$ and $\alpha_{2}$$\left(0\leq \alpha_{1},\alpha_{2}<1\right)$ are empirical broadening parameters that account for a distribution of relaxation times. In physical terms, non-zero α values indicate that the corresponding relaxation process is no longer governed by a single well-defined timescale but rather by a heterogeneous dynamic environment in which correlated interactions, local disorder, clustering, or structural heterogeneity broaden the dielectric response [22]. The Debye model is recovered as the limiting case $\alpha_{1}=\alpha_{2}=0$.

This generalized description is particularly appropriate for the present system. Water and acetone differ markedly in polarity, hydrogen bonding capability, and molecular polarizability ($P_{water}\approx 3.8\pm 0.03\;C\;\cdot \;m^{-2}$; $P_{acetone}\approx 7.9\pm 0.2\;C\;\cdot \;m^{-2}$) [23]. Water and acetone differ markedly in polarity, hydrogen-bonding capability, and molecular polarizability. For the latter quantity, reported THz values are $3.81\pm 0.03\;{Å}^{3}$ for water and $7.9\pm 0.2\;{Å}^{3}$ for acetone [23]. Therefore, their mixture cannot be treated as a simple non-interacting combination of the two pure liquids. In addition to self-correlations within each component, the dielectric response of the mixture is influenced by water–acetone hydrogen bonding, dipole–dipole interactions, and local compositional heterogeneity. Within the Cole–Cole framework, the dielectric strengths $\Delta \varepsilon_{1}$ and $\Delta \varepsilon_{2}$ can be related to effective orientational correlations through Kirkwood–Fröhlich (KF) factors $g_{K1}$ and $g_{K2}$ [24], according to

(3) $$\Delta \varepsilon_{1(2)}=g_{K1(2)}\frac{N\mu_{\mathrm{eff}}^{2}}{3\varepsilon_{0}k_{B}T}$$

where *N* is the dipole density, and $\mu_{\mathrm{eff}}$ is the effective dipole moment.

In pure liquids, $g_{K}>1$ indicates a net tendency toward parallel dipolar correlations, whereas $g_{K}<1$ predominantly reflects antiparallel correlations; $g_{K}=1$ can be phenomenologically associated with uncorrelated dipoles [25]. In binary mixtures, however, the corresponding quantity should be regarded as an effective, composition-dependent parameter embedding both like–like and unlike orientational correlations [26].

**Figure 1:**
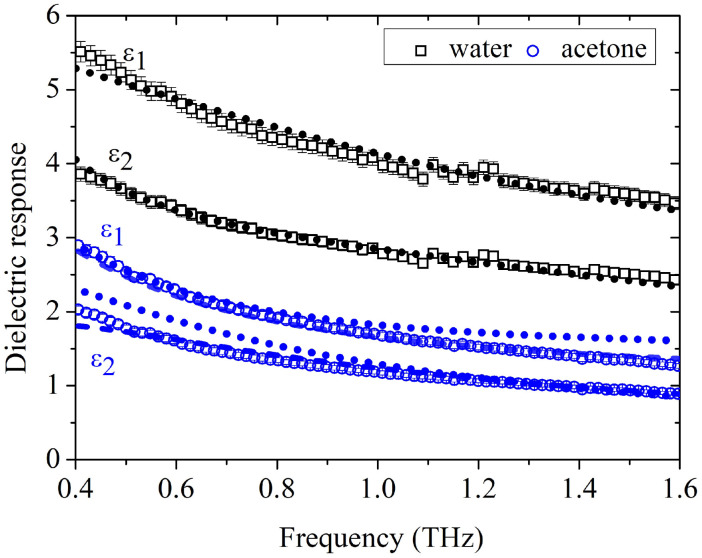
Measured complex dielectric function of pure water and pure acetone (symbols) as a function of frequency, together with the best fits obtained using the double Debye model (dotted lines) and the double Cole–Cole model (dashed lines).

Figure 1 shows the experimentally retrieved complex dielectric function, $\tilde{\varepsilon }\left(\omega \right)=\varepsilon_{1}\left(\omega \right)+i\varepsilon_{2}\left(\omega \right)$, of pure water and pure acetone in the 0.4–1.6 THz range, together with the best fits obtained using the double Debye and double Cole–Cole models. The fitting procedure was based on nonlinear regression simultaneously performed on the real and imaginary parts of the dielectric response, using $\Delta \varepsilon_{1}$, $\Delta \varepsilon_{2}$, $\tau_{1}$, $\tau_{2}$, and, for the Cole–Cole model, $\alpha_{1}$ and $\alpha_{2}$ as free parameters. The high-frequency permittivity $\varepsilon_{\infty }$ was initialized from the high-frequency behavior of the experimental $\varepsilon_{1}\left(\omega \right)$ data.

As shown in Figure 1, the double Debye model accurately reproduces the dielectric response of pure water, consistent with its nearly Debye-like behavior in the investigated spectral range.

By contrast, the same model provides only a partial description of pure acetone, whereas the double Cole–Cole expression yields a consistent fit for both liquids. In the case of water, the Cole–Cole model naturally collapses to the Debye limit, with $\alpha_{1}=\alpha_{2}=0$, while for acetone, finite broadening parameters are required. The fitting parameters obtained from the Cole–Cole analysis are summarized in Table 1, together with the corresponding goodness-of-fit metrics to further assess the robustness of the nonlinear regression, represented by the normalized root mean square error (NRMSE). The extracted values are in good agreement with the literature data [18,27] and support the use of the double Cole–Cole formalism as a unified framework for describing both pure liquids and intermediate mixtures. For clarity, a direct comparison between the fitted parameters of the pure liquids and the representative literature values [20,28,29] is provided in the same table.

Although the slow relaxation process of both water and acetone lies mainly below the directly covered spectral window, the extracted $\tau_{1}$ values remain fully consistent with the established literature data and with the global fit of the complex dielectric response. The present measurements therefore do not directly resolve the full low-frequency maximum of the slow mode, but they still provide a physically meaningful estimate of its characteristic timescale within the adopted phenomenological framework.

After validating the fitting strategy on the two pure liquids, we extended the analysis to 15 water–acetone mixtures distributed over the full composition range. For each sample, the complex dielectric response was measured as a function of frequency and fitted using the double Cole–Cole model. In this sense, the adopted model should be viewed as an effective phenomenological description of the mixture’s dynamics, consistently anchored to the independently measured responses of pure water and pure acetone, whose fitted parameters are in good agreement with the literature values. While not implying a unique microscopic decomposition of the underlying molecular dynamics, the model nevertheless provides a physically motivated description of the concentration-dependent dielectric response of the mixtures, in line with the approaches in the literature in which interacting binary polar liquids are described through effective collective relaxation channels rather than through a direct superposition of all channels of the isolated pure components [18,20].

**Figure 2:**
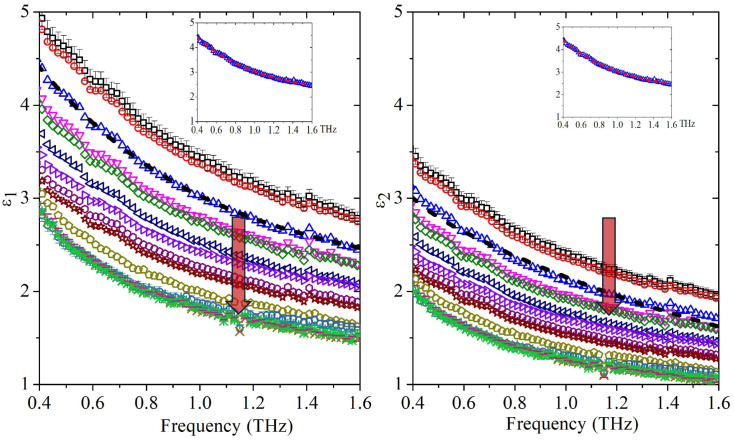
Measured real $\varepsilon_{1}$ and imaginary $\varepsilon_{2}$ components of the dielectric function of the water–acetone mixtures as a function of frequency for increasing acetone molar fractions (from top to bottom). In the inset, the experimental data and the corresponding double Cole–Cole fit are shown for the representative composition $X_{M}=4\%$.

Figure 2 show the frequency-dependent real and imaginary parts of the dielectric function, respectively, for all investigated mixtures. In both plots, $\varepsilon_{1}\left(\omega \right)$ and $\varepsilon_{2}\left(\omega \right)$ decrease monotonically with increasing frequency over the whole concentration range. As the acetone content increases, the dielectric response progressively shifts toward lower values, consistent with the lower static permittivity and reduced absorption of acetone compared with water. In the inset, the fit (dashed curve) obtained for the 4% molar fraction together with the experimental data is shown as a representative example, illustrating the ability of the double Cole–Cole model to reproduce the measured spectral broadening. The fitting parameters using the nonlinear regression procedure on both $\varepsilon_{1}$ and $\varepsilon_{2}$ are listed in Table S1 in the Supplementary Material.

**Figure 3:**
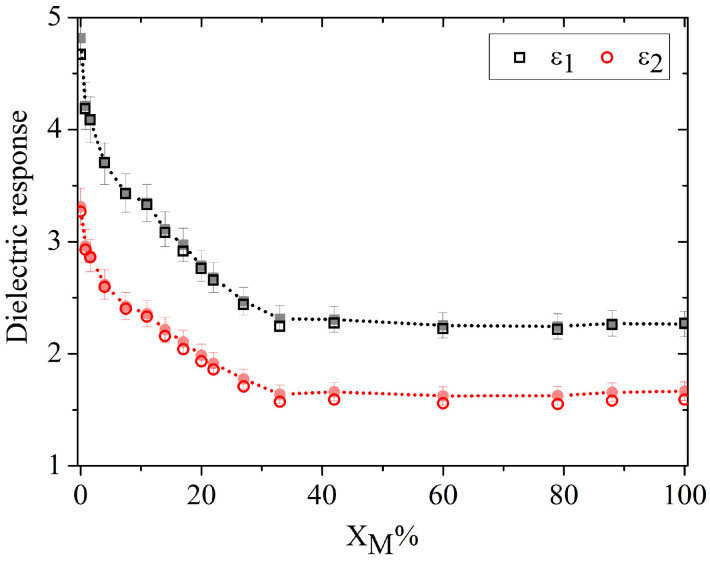
The experimental dielectric response at 0.6 THz as a function of the acetone molar fraction $X_{M}$ (open symbols), together with the corresponding values obtained from the double Cole–Cole fit (filled symbols), for both the real $\varepsilon_{1}$ and imaginary $\varepsilon_{2}$ parts of the dielectric function. Dotted lines are guides for the eye.

To better visualize the concentration dependence of the dielectric response, Figure 3 reports the experimental values for $\varepsilon_{1}$ and $\varepsilon_{2}$ at 0.6 THz (open symbols), together with the corresponding values obtained from the fitting procedure (filled symbols). This frequency was selected because it provided the highest signal-to-noise ratio within the investigated spectral window. The comparison shows excellent agreement between the experiment and model over the whole molar fraction range, confirming the robustness of the fitting procedure. A marked and strongly nonlinear decrease in both $\varepsilon_{1}$ and $\varepsilon_{2}$ was already observed at very low acetone contents, with measurable variations appearing for concentrations as low as 0.8 mol%. This behavior reflects the abrupt perturbation of the extended hydrogen bond network of bulk water induced by the addition of an aprotic hydrogen bond acceptor [3,5,6]. The pronounced initial slope of both dielectric components in the water-rich region also explains the particularly high sensitivity of the method at low solute concentrations.

The uncertainty associated with the ATR-TDS extraction of the dielectric function is approximately 5%. On this basis, the limit of detection (LOD) for acetone can be estimated as

(4) $$\mathrm{LOD}=\frac{3\sigma_{\varepsilon_{1(2)}}}{d\varepsilon_{1(2)}/dX_{M}}$$

where $\sigma_{\varepsilon_{1(2)}}=0.05\;\varepsilon_{1(2)}$, and $d\varepsilon_{1(2)}/dX_{M}$ is the local slope of the $\varepsilon_{1}\left(X_{M}\right)$ and $\varepsilon_{2}\left(X_{M}\right)$ curves. In the water-rich region, where the concentration dependence is steepest, the estimated LOD is approximately 0.5% from $\varepsilon_{1}$ and 0.6% from $\varepsilon_{2}$. These values indicate that ATR-TDS is highly sensitive to small perturbations in hydrogen bond connectivity and local dipolar organization, making it particularly effective for detecting acetone in aqueous environments at low concentrations.

**Figure 4:**
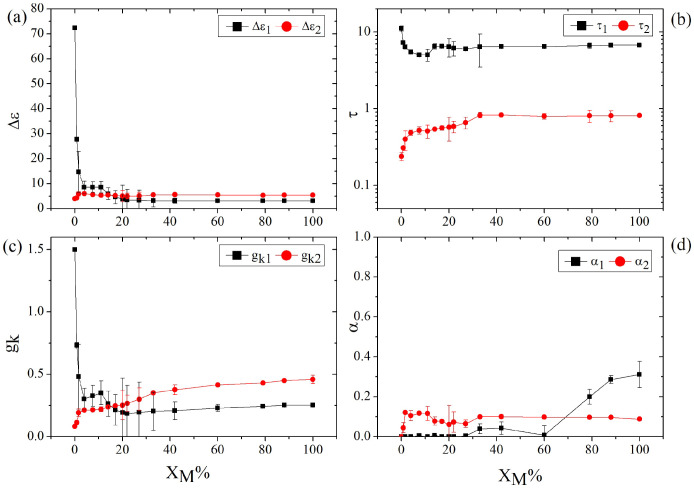
Parameters extracted from the double Cole–Cole analysis as a function of acetone molar fraction $X_{M}$: (**a**) dielectric strengths $\Delta \varepsilon_{1}$ and $\Delta \varepsilon_{2}$; (**b**) slow and fast relaxation times, $\tau_{1}$ and $\tau_{2}$ (semilog scale); (**c**) effective Kirkwood–Fröhlich correlation factors, $g_{K1}$ and $g_{K2}$; (**d**) broadening parameters $\alpha_{1}$ and $\alpha_{2}$.

While the concentration dependence of $\varepsilon_{1}$ and $\varepsilon_{2}$ reveals the strong sensitivity of the THz response to acetone addition, a more detailed physical interpretation requires an analysis of the parameters extracted from the double Cole–Cole fits. Figure 4 summarizes the parameters extracted from the double Cole–Cole analysis for all investigated mixtures as a function of the acetone molar fraction $X_{M}$. In addition to the dielectric strengths $\Delta \varepsilon_{1}$ and $\Delta \varepsilon_{2}$, the corresponding relaxation times $\tau_{1}$ and $\tau_{2}$, and the broadening parameters $\alpha_{1}$ and $\alpha_{2}$, the figure also reports the effective Kirkwood–Fröhlich correlation factors $g_{K1}$ and $g_{K2}$ calculated from the retrieved dielectric strengths using Equation (3). As already mentioned, since the double Cole–Cole model contains several adjustable parameters relative to the available frequency window, it should be regarded here as an effective minimal model chosen for its ability to consistently reproduce the measured spectra across the full composition range.

The behavior of the dielectric strengths, shown in Figure 4a, immediately reveals the markedly different sensitivities of the two relaxation channels to acetone addition. Even the smallest molar fraction of acetone produces a sharp decrease in $\Delta \varepsilon_{1}$, whereas $\Delta \varepsilon_{2}$ remains nearly constant over the whole concentration range. The strong suppression of the slow dielectric strength indicates that acetone primarily disrupts the cooperative reorganization of the extended hydrogen bond network of water. In pure water, the slow relaxation process, characterized by $\tau_{1}$ in the order of 10 ps, is generally associated with large-scale collective orientational rearrangements involving strongly correlated molecules within the hydrogen bond network [19,20]. Its dielectric intensity therefore depends critically on the continuity and cooperativity of the water–water hydrogen bond structure. The addition of acetone, an aprotic molecule that can accept but not donate hydrogen bonds, perturbs this network by interrupting its three-dimensional connectivity and reducing the number of configurations able to sustain such collective relaxation [3,5,6]. As a consequence, the dielectric strength of the slow mode decreases sharply as the acetone content increases. By contrast, the faster relaxation channel, associated with $\tau_{2}$ in the order of 0.1 ps, appears much less sensitive to the presence of acetone. This suggests that the fast process is governed by more local intermolecular dynamics, such as libration-assisted orientational fluctuations, transient hydrogen bond rearrangements, or short-range cage motions, which do not rely as strongly on the existence of an extended and percolating hydrogen bond network [19,20].

The corresponding relaxation times are reported in Figure 4b on a semilog scale for the sake of clarity. As already discussed for the case of pure water and pure acetone, the absolute values of the slow relaxation time must be interpreted with caution, and the detailed concentration dependence of $\tau_{1}$, especially in the water-rich region, should not be over-interpreted. In this regime, part of the local variation may also reflect parameter correlation and the effective nature of the adopted phenomenological model within the available spectral window.

The overall emerging picture for both $\tau_{1}$ and $\tau_{2}$ is that only a relatively weak dependence on acetone concentration is observed. This suggests that the addition of the aprotic solute does not primarily modify the intrinsic timescale of the underlying molecular motions. Rather, acetone acts mainly by reducing the statistical weight and cooperativity of the relaxing environments, especially those associated with the slow hydrogen-bond-driven process. In this picture, the spatial continuity of the water network is progressively disrupted, thereby suppressing the amplitude of the slow mode without substantially altering the characteristic relaxation time of the residual water-rich structures that are still able to sustain it. A similar argument applies to the fast relaxation channel: if this process originates from local motions, such as libration-assisted fluctuations or short-range hydrogen bond rearrangements, its characteristic timescale may remain close to that of pure water even in the presence of significant structural perturbation [19,20].

Figure 4c reports the effective Kirkwood–Fröhlich factors $g_{K1}$ and $g_{K2}$. Because these correlation factors are directly proportional to the dielectric strengths of the two relaxation channels, their compositional evolution largely follows that of $\Delta \varepsilon_{1}$ and $\Delta \varepsilon_{2}$. In the present case, however, the most informative aspect is not simply their variation with acetone molar fraction but rather the absolute values they assume. Within the standard Kirkwood–Fröhlich framework, values greater than unity are generally associated with predominantly parallel dipolar correlations, whereas values below unity suggest a tendency toward antiparallel orientational arrangements [25]. Strictly speaking, this interpretation is rigorously defined for pure liquids; nevertheless, when applied with due caution, it can still provide useful qualitative insight into the local dipolar organization of binary mixtures [26]. From this perspective, the value of $g_{K1}$ close to 2 in pure water is fully consistent with the well-established picture of a highly correlated hydrogen bond network and agrees well with values reported in classical molecular dynamics studies at room temperature [30]. The sharp decrease in $g_{K1}$ observed upon the first addition of acetone points to a rapid breakdown of the cooperative dipolar correlations associated with the slow relaxation channel. At intermediate compositions, the more gradual variation in $g_{K1}$ suggests that some hydrogen-bonded water motifs may still survive, although in a progressively more fragmented and less cooperative environment. At higher acetone contents, where a continuous water network can no longer be maintained, the fact that $g_{K1}$ remains below unity indicates that the residual dipolar correlations are qualitatively different from those of pure water and may reflect a more locally compensated and weakly cooperative arrangement. A similar argument applies to $g_{K2}$, whose values remain below unity over the whole concentration range. Although any structural interpretation must remain qualitative in a mixed liquid, this behavior suggests that the fast relaxation channel is associated with local molecular configurations that do not exhibit strong cooperative alignment and may instead favor partial dipolar compensation. In this sense, the persistently low values of the corresponding correlation factor are compatible with the presence of small water aggregates or locally compensated dipolar configurations, in which local dipole compensation is statistically favored [31,32].

This distinction is further supported by the behavior of the broadening parameters, shown in Figure 4d, which provide complementary information on the dynamic heterogeneity of the same two relaxation channels. The parameter $\alpha_{2}$ becomes non-zero immediately after the addition of acetone and rapidly saturates around 0.1, indicating that the fast relaxation channel is highly sensitive to the onset of local heterogeneity, although its ultrafast dynamics remain distributed over a relatively narrow range of time scales [22]. By contrast, $\alpha_{1}$ remains essentially zero, within experimental uncertainty, up to $X_{M}\approx 0.6$, and increases only at higher acetone concentrations. This delayed broadening suggests that the slow relaxation process retains an almost Debye-like character as long as residual water-rich regions are still able to sustain collective hydrogen bond network dynamics. In this regime, acetone mainly reduces the dielectric strength of the slow mode without substantially broadening its time distribution. Only when the extended water network is no longer maintained does a significant distribution of slow relaxation times emerge.

Overall, the concentration dependence of the dielectric strengths, relaxation times, effective correlation factors, and broadening parameters converges toward a coherent molecular picture: acetone acts primarily as a network breaker, strongly suppressing the cooperative hydrogen bond network of water while leaving the faster and more local relaxation channel comparatively less affected. The THz response of the mixtures is therefore governed not by a simple interpolation between the two pure liquids but by the reorganization of hydrogen bond connectivity, which is naturally concentration-dependent, by dipolar correlations, and by local dynamical heterogeneity.

## 3. Materials and Methods

### 3.1. Sample Preparation

Binary water–acetone mixtures were prepared using acetone (99.7%, ROMIL Ltd., Waterbeach, UK) and deionized Milli-Q water. Mixtures at the desired compositions were obtained by combining appropriate volumes of acetone and water with a volumetric precision of 0.05 mL, covering the full range from pure water to pure acetone.

The compositions were initially defined in terms of volume fraction and then converted into acetone molar fraction, $X_{M}$, using the molar masses of water ($M_{H_{2}O}=18.015\;g\;{\mathrm{mol}}^{-1}$) and acetone ($M_{\mathrm{acetone}}=58.08\;g\;{\mathrm{mol}}^{-1}$). The investigated mixtures were selected so as to cover three composition regimes: (i) a water-rich region ($X_{M}=0.8,1.6,4,7.5,11,14,17,20,$ and 22%), (ii) an intermediate-concentration region ($X_{M}=27,33,$ and 42%), and (iii) an acetone-rich region ($X_{M}=60,79,$ and 88%), in addition to the two pure liquids.

All samples were prepared and stored in sealed containers at room temperature ($25^{\;\circ }$C) in order to minimize evaporation prior to the THz measurements.

### 3.2. Experimental Setup

The THz measurements were performed by combining a commercial terahertz time-domain spectroscopy system (TERA K15, Menlo Systems, Planegg, Germany) with an attenuated total reflection (ATR) module based on a silicon prism (BATOP GmbH, Jena, Germany). This configuration was specifically selected for the investigation of water–acetone mixtures because it offers major advantages for the analysis of strongly absorbing and volatile liquid systems in the THz range.

In the ATR geometry, the sample is probed through the evanescent field generated at the prism–sample interface under total internal reflection conditions [14] rather than through direct propagation across a finite liquid thickness. This substantially reduces the effective interaction length and makes it possible to investigate highly absorbing aqueous media in a spectral region where conventional transmission measurements would be severely limited [12]. In addition, the ATR configuration minimizes uncertainties associated with sample thickness and liquid-layer uniformity, which are often critical in transmission-based measurements of liquids.

In the present case, the ATR approach also provided a major practical advantage in terms of sample stability. Because acetone is highly volatile, evaporation during acquisition may alter the actual composition of the mixture, especially at low acetone concentrations where even small losses of solute can produce measurable changes in the dielectric response. The ATR arrangement adopted here allowed the liquid sample to be measured in a controlled and nearly closed environment, thereby substantially reducing evaporation-induced compositional changes during the experiment. This aspect was particularly important for ensuring reliable measurements over the whole concentration range and for preserving sensitivity in the water-rich regime.

**Figure 5:**
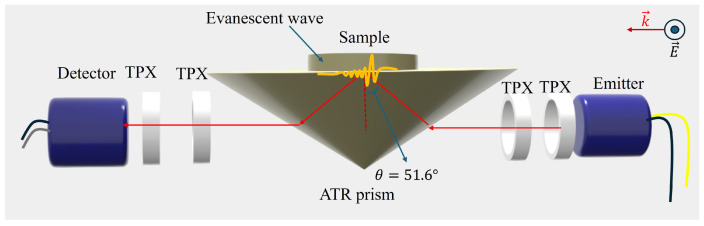
Schematic representation of the ATR-TDS setup used in this work, including the silicon prism, the sealed liquid pool, and the incidence geometry of the *s*-polarized THz beam.

Figure 5 shows a schematic representation of the THz ATR-TDS system used in this work. The focused s-polarized beam impinged on the silicon prism at an incidence angle of $\theta =51.6^{\circ }$. Under these conditions, the setup was suitable for the investigation of materials with refractive index lower than approximately 2.5, for which total internal reflection was ensured at the prism–sample interface. The liquid sample was placed in a sealed pool ($40\times 25\times 10\;{\mathrm{mm}}^{3}$) mechanically coupled to the prism and covered with a vacuum cap during the measurement in order to limit evaporation. After each acquisition, the pool was carefully cleaned before introducing the next solution.

Overall, the combination of the silicon ATR prism with the THz TDS platform enabled sensitive and reproducible measurements of water–acetone mixtures over the full concentration range, while maintaining good control over sample handling and compositional stability. At the same time, the method remained intrinsically sensitive to optical alignment and to the exact measurement geometry, so that particular care had to be taken in beam positioning, prism–sample coupling conditions, and the reproducibility of the sample–prism contact [33].

### 3.3. Dielectric Response Retrieval

Because THz time-domain spectroscopy is a coherent detection technique, both the amplitude and the phase of the reflected electric field are experimentally accessible. This makes it possible to determine the complex optical response of the sample directly rather than inferring it from intensity measurements alone. The reflected electric field at the prism–sample interface was analyzed within the Fresnel formalism for *s*-polarized radiation [34]. Under total internal reflection conditions, the complex reflection coefficient $\tilde{r_{s}}$ depends on the incidence angle and the complex refractive index $\tilde{n}=n+ik$ of the sample through the following equation [34]:

(5) $$\tilde{r_{s}}=\frac{n_{1}\cos\theta -\tilde{n}\sqrt{1-{\left(\frac{n_{1}}{\tilde{n}}\sin\theta \right)}^{2}}}{n_{1}\cos\theta +\tilde{n}\sqrt{1-{\left(\frac{n_{1}}{\tilde{n}}\sin\theta \right)}^{2}}}\;$$

where $n_{1}=3.4$ as the THz beam propagates through the silicon prism before interacting with the sample.

By comparing the reflected signal measured from the liquid with the corresponding reference signal, the complex dielectric function $\tilde{\varepsilon }=\varepsilon_{1}+i\varepsilon_{2}$ of the sample was retrieved as a function of acetone’s molar fraction and over the investigated frequency range, since $\tilde{\varepsilon }={\tilde{n}}^{2}$.

In the ATR configuration, the interaction with the liquid occurs through the evanescent field penetrating the sample from the prism surface. The corresponding penetration depth depends on the wavelength, the incidence angle, and the refractive indices of the prism and sample [35]:

(6) $$d_{p}=\frac{\lambda }{2\pi n_{1}\sqrt{\sin^{2}\left(\theta \right)-{\left(\frac{n}{n_{1}}\right)}^{2}}}$$

For the present geometry, the estimated penetration depth at 0.6 THz is approximately 26μm for pure water and 18.5μm for pure acetone, confirming that the measurement probes only a shallow interfacial region of the liquid while remaining fully sensitive to its dielectric response.

## 4. Conclusions

In this work, terahertz time-domain spectroscopy in an attenuated total reflection configuration (ATR-TDS) was employed to investigate the dielectric response and intermolecular dynamics of water–acetone mixtures over the full molar concentration range. The measurements reveal a strongly nonlinear evolution of the THz dielectric function with composition, reflecting the progressive transformation of the liquid from a highly cooperative hydrogen-bonded water network to an acetone-rich environment dominated by weaker and more local dipolar interactions.

The strongest spectral sensitivity is observed in the water-rich region, where even small amounts of acetone induce a pronounced modification of both the real and imaginary parts of the dielectric function. This behavior is consistent with the rapid disruption of the extended hydrogen bond network of water and with the associated loss of cooperative dipolar correlations. At intermediate compositions, the system evolves toward a structurally heterogeneous solvation environment, whereas in the acetone-rich regime, the dielectric response becomes much less concentration-dependent, indicating that the extended water network can no longer be sustained.

The double Cole–Cole analysis provides a coherent molecular interpretation of this evolution. The strong suppression of the slow dielectric strength, together with the decrease in the corresponding effective Kirkwood–Fröhlich factor, is consistent with assigning the slow relaxation channel as the one most directly associated with collective hydrogen bond network dynamics. By contrast, the fast relaxation channel remains comparatively less affected in both dielectric strength and characteristic time, supporting its assignment to more local intermolecular motions. The behavior of the broadening parameters shows instead that local heterogeneity emerges immediately in the fast channel, whereas significant broadening of the slow mode appears only when the water network has become largely fragmented.

Overall, the present results suggest that the THz response of water–acetone mixtures is governed not simply by the intrinsic relaxation properties of the pure components but by the progressive reorganization of hydrogen bond connectivity, dipolar correlations, and local dynamical heterogeneity across the whole composition range. THz ATR-TDS therefore emerges as a sensitive tool for probing subtle intermolecular rearrangements in polar liquid mixtures and provides a physically grounded framework for the detection of acetone and other hydrogen-bond-active volatile species in aqueous environments.

## Figures and Tables

**Table 1:** Fitting parameters of pure water and acetone using the double Cole–Cole model. Values from the literature are included for comparison.

Liquid	$\varepsilon_{\infty }$	$\tau_{1}$ (ps)	$\tau_{2}$ (ps)	$\Delta \varepsilon_{1}$	$\Delta \varepsilon_{2}$	$\alpha_{1}$	$\alpha_{2}$	NRMSE	Ref.
Water	3.0 ± 0.2	11.2 ± 0.9	0.20 ± 0.03	72.5 ± 0.1	3.3 ± 0.1	0	0	0.03	This work
	3.48	8.24	0.181	73.38	1.5	–	–		[20]
Acetone	1.3 ± 0.1	6.7 ± 0.5	0.8 ± 0.4	3.1 ± 0.1	5.4 ± 0.5	0.3 ± 0.1	0.10 ± 0.01	0.03	This work
	–	≈3.5	≈1.2	–	–	–	–		[28,29]

## Data Availability

The original contributions presented in this study are included in this article. Further inquiries can be directed to the corresponding authors.

## Supplementary Materials

The following supporting information can be downloaded at: https://www.mdpi.com/article/10.3390/ijms27125188/s1.
