# Supplementary material for: THz ATR-TDS Spectroscopy of Acetone–Water Mixtures: Hydrogen Bonding to Dipole–Dipole Dynamics
Source: Int J Mol Sci. 2026 Jun 8;27(12):5188. doi: 10.3390/ijms27125188 (PMC13299297; doi:10.3390/ijms27125188)
Supplement: Supplementary file 1 [file ijms-27-05188-s001.zip › ijms-4307632-supplementary.pdf]

Supplementary Material

Table S1. Fitting parameters obtained using the Cole-Cole model and goodness-of-fit evaluated using the Normalized Root Mean Square Error (NRMSE) for the water-acetone mixture at different acetone molar concentrations X_M.

X_{M} **(%)**	$\Delta\varepsilon_1$	$\Delta\varepsilon_2$	τ_1 **(ps)**	τ_2 **(ps)**	**gK$_1$**	**gK$_2$**	α_1	α_2	**NRMSE**
0.8	27.8 ± 0.5	4.2 ± 0.2	7.1 ± 0.6	0.30 ± 0.04	0.72 ± 0.05	0.14 ± 0.02	0.00 ± 0.01	0.12 ± 0.02	0.02
1.6	15 ± 4	5.7 ± 0.3	6.6 ± 0.5	0.38 ± 0.05	0.5 ± 0.2	0.20 ± 0.03	0.00 ± 0.01	0.11 ± 0.02	0.01
4	8 ± 2	6.2 ± 0.4	5.6 ± 0.6	0.48 ± 0.05	0.30 ± 0.1	0.20 ± 0.04	0.00 ± 0.01	0.10 ± 0.03	0.03
7.5	8 ± 2	6.0 ± 0.4	5.5 ± 0.6	0.52 ± 0.05	0.3 ± 0.1	0.21 ± 0.04	0.00 ± 0.01	0.11 ± 0.03	0.03
11	8.5 ± 0.4	6.0 ± 0.4	5.0 ± 0.6	0.54 ± 0.05	0.3 ± 0.1	0.22 ± 0.04	0.00 ± 0.01	0.12 ± 0.03	0.05
14	8 ± 1	5.8 ± 0.5	5.5 ± 0.7	0.55 ± 0.06	0.3 ± 0.1	0.22 ± 0.04	0.00 ± 0.01	0.09 ± 0.03	0.04
17	5.0 ± 0.4	5.7 ± 0.5	5.9 ± 0.8	0.58 ± 0.06	0.3 ± 0.1	0.22 ± 0.05	0.00 ± 0.01	0.08 ± 0.03	0.06
20	3.9 ± 0.4	5.5 ± 0.5	5.9 ± 0.9	0.60 ± 0.07	0.3 ± 0.1	0.24 ± 0.05	0.00 ± 0.01	0.07 ± 0.03	0.05
22	3 ± 1	5.4 ± 0.5	6.2 ± 0.7	0.58 ± 0.07	0.2 ± 0.1	0.24 ± 0.05	0.00 ± 0.01	0.06 ± 0.03	0.05
27	3 ± 1	5.2 ± 0.4	6.0 ± 0.4	0.64 ± 0.08	0.2 ± 0.1	0.26 ± 0.05	0.00 ± 0.01	0.07 ± 0.03	0.04
33	3.3 ± 0.8	5.3 ± 0.4	6 ± 2	0.70 ± 0.08	0.20 ± 0.09	0.25 ± 0.05	0.00 ± 0.02	0.06 ± 0.03	0.05
42	3.1 ± 0.5	5.5 ± 0.3	6.0 ± 0.7	0.82 ± 0.05	0.18 ± 0.07	0.31 ± 0.04	0.03 ± 0.02	0.10 ± 0.02	0.03
60	3.1 ± 0.1	5.4 ± 0.3	6.1 ± 0.6	0.82 ± 0.05	0.20 ± 0.05	0.36 ± 0.03	0.04 ± 0.02	0.10 ± 0.02	0.03
79	3.1 ± 0.1	5.4 ± 0.3	6.1 ± 0.5	0.78 ± 0.05	0.22 ± 0.04	0.41 ± 0.03	0.20 ± 0.02	0.10 ± 0.02	0.03
88	3.1 ± 0.1	5.4 ± 0.3	6.2 ± 0.5	0.80 ± 0.05	0.24 ± 0.04	0.42 ± 0.03	0.27 ± 0.03	0.10 ± 0.02	0.03
